# Supplementary material for: Effect of the Interaction between Elevated Carbon Dioxide and Iron Limitation on Proteomic Profiling of Soybean
Source: Int J Mol Sci. 2022 Nov 7;23(21):13632. doi: 10.3390/ijms232113632 (PMC9654904; doi:10.3390/ijms232113632)
Supplement: Supplementary file 1 [file ijms-23-13632-s001.zip › Supplementary Material_figures.pdf]

Supplementary Materials:

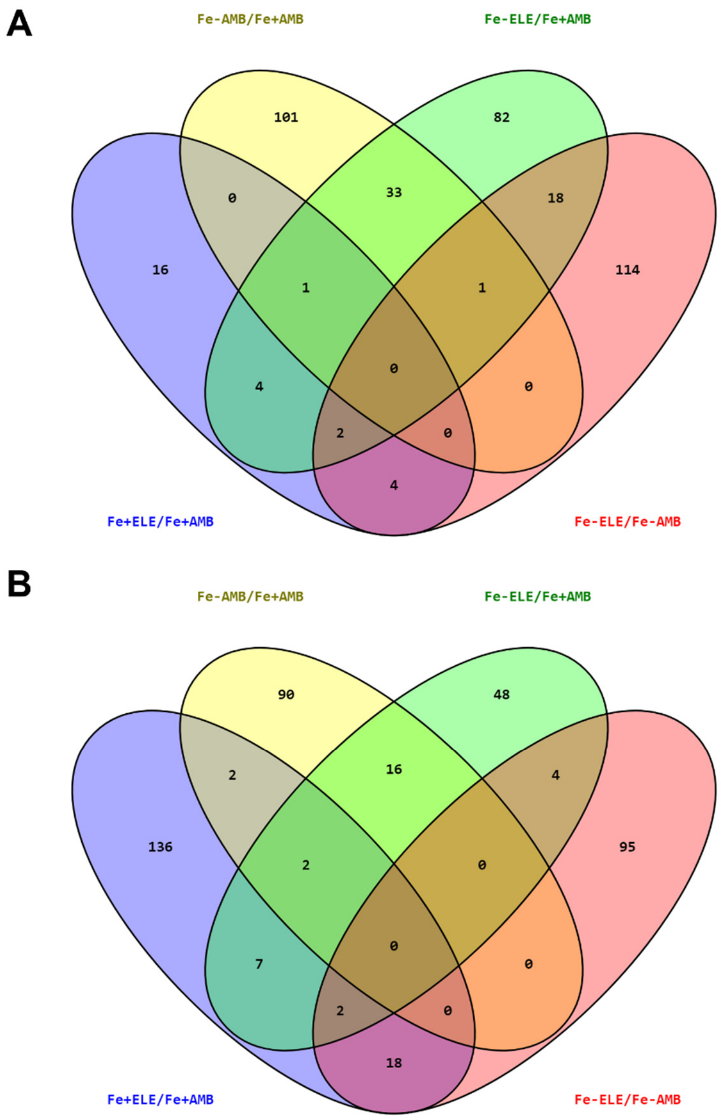

Figure S1. Venn diagram significantly displaying (a) upregulated and (b) downregulated proteins in response to eCO_2 and Fe-limitation in roots. Fe+AMB, Fe-sufficient + aCO_2; Fe+ELE, Fe-sufficient + eCO_2; Fe-AMB, Fe-limitation + aCO_2; Fe-ELE, Fe-limitation + eCO_2.

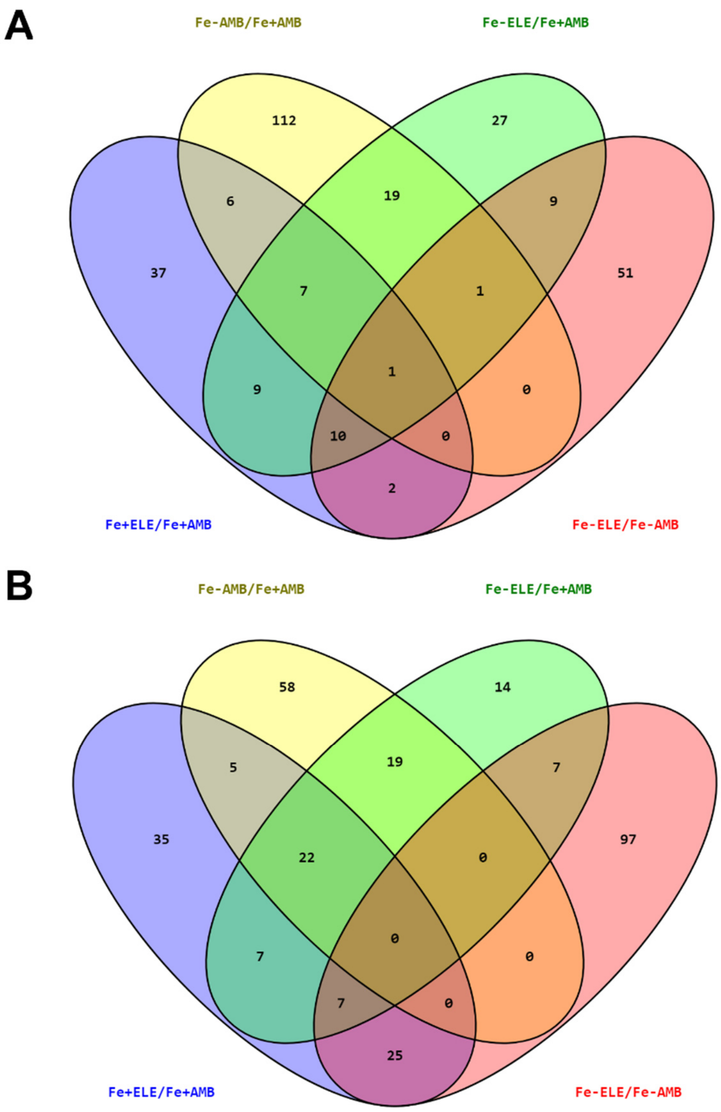

Figure S2. Venn diagram significantly displaying (a) upregulated and (b) downregulated proteins in response to eCO_2 and Fe-limitation in leaves. Fe+AMB, Fe-sufficient + aCO_2; Fe+ELE, Fe-sufficient + eCO_2; Fe-AMB, Fe-limitation + aCO_2; Fe-ELE, Fe-limitation + eCO_2.

A

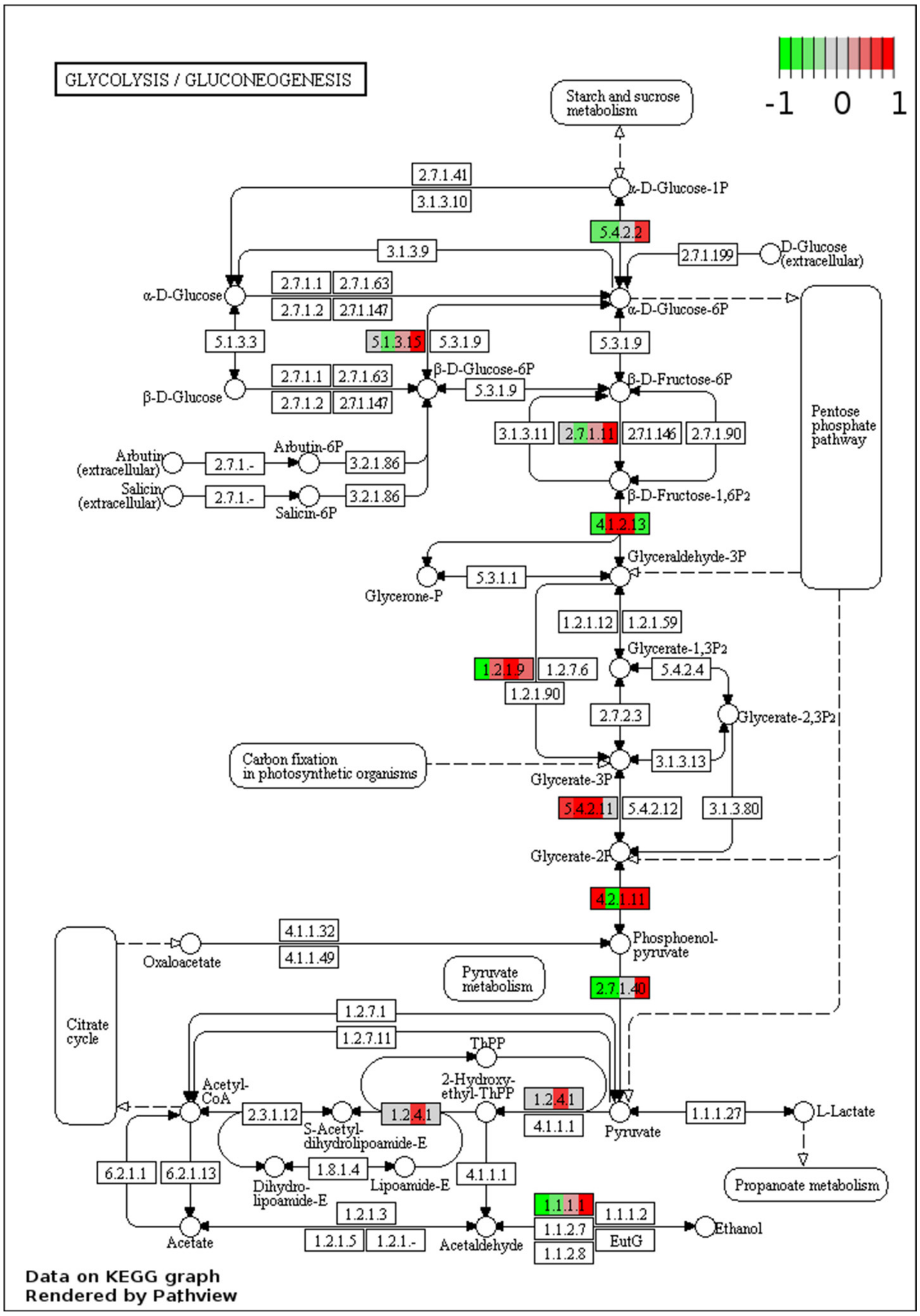
GLYCOLYSIS / GLUCONEOGENESIS
-1 0 1
Starch and sucrose metabolism
α-D-Glucose-1P
2.7.1.41
3.1.3.10
5.4.2.2
3.1.3.9
2.7.1.199
D-Glucose (extracellular)
α-D-Glucose
2.7.1.1
2.7.1.63
2.7.1.2
2.7.1.147
α-D-Glucose-6P
5.1.3.3
5.1.3.15
5.3.1.9
β-D-Glucose
β-D-Glucose-6P
β-D-Fructose-6P
Pentose phosphate pathway
3.1.3.11
2.7.1.11
2.7.1.146
2.7.1.90
Arbutin (extracellular)
2.7.1.-
Arbutin-6P
3.2.1.86
Salicin (extracellular)
Salicin-6P
β-D-Fructose-1,6P2
4.1.2.13
Glyceraldehyde-3P
Glycerone-P
5.3.1.1
1.2.1.12
1.2.1.59
Glycerate-1,3P2
1.2.1.9
1.2.7.6
1.2.1.90
5.4.2.4
Glycerate-2,3P2
2.7.2.3
Carbon fixation in photosynthetic organisms
Glycerate-3P
3.1.3.13
5.4.2.11
5.4.2.12
3.1.3.80
Glycerate-2P
4.2.1.11
Citrate cycle
Oxaloacetate
4.1.1.32
4.1.1.49
Phosphoenol-pyruvate
Pyruvate metabolism
2.7.1.40
1.2.7.1
1.2.7.11
ThPP
2-Hydroxy-ethyl-ThPP
Acetyl-CoA
2.3.1.12
S-Acetyl-dihydrolipoamide-E
1.2.4.1
4.1.1.1
Pyruvate
1.1.1.27
L-Lactate
Propanoate metabolism
6.2.1.1
6.2.1.13
Dihydro-lipoamide-E
1.8.1.4
Lipoamide-E
4.1.1.1
1.2.1.3
1.2.1.5
1.2.1.-
1.1.1.1
1.1.1.2
1.1.2.7
EutG
1.1.2.8
Acetate
Acetaldehyde
Ethanol
Data on KEGG graph
Rendered by Pathview

B

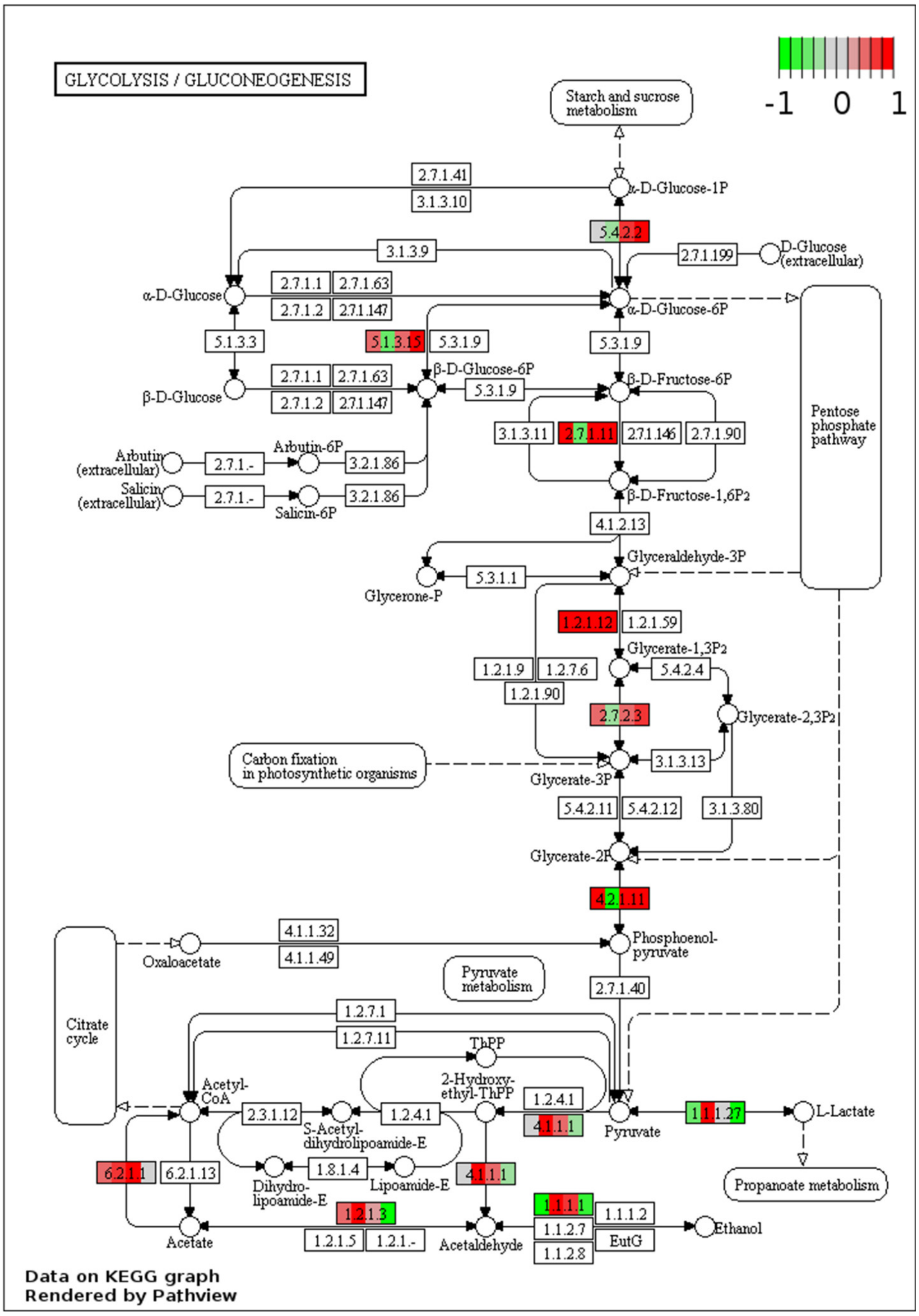

Figure S3. Differential transcript expression in the 'glycolysis/gluconeogenesis' KEGG pathway in the response to eCO_2 and Fe- limitation in root (a), and leaf tissues (b). Each enzyme contains four color bars indicating the mean expression of Fe+ELE/Fe+AMB (first), Fe-AMB/Fe+AMB (second), Fe-ELE/Fe+AMB (third), and Fe-ELE/Fe-AMB (fourth) treatments. Red color indicating increased expression, and green indicating decreased expression. 1.1.1.1 - alcohol dehydrogenase; 1.1.1.27 - L-lactate dehydrogenase; 1.2.1.3 - aldehyde dehydrogenase; 1.2.1.9 - NADP-dependent glyceraldehyde-3-phosphate dehydrogenase; 1.2.1.12 - glyceraldehyde 3-phosphate dehydrogenase; 2.7.1.11 - ATP-dependent 6-phosphofructokinase; 2.7.1.40 - pyruvate kinase; 2.7.2.3 - phosphoglycerate kinase; 4.1.1.1 - pyruvate decarboxylase; 4.1.2.13 - fructose-bisphosphate aldolase; 4.2.1.11 – enolase; 5.1.3.15 - glucose-6-phosphate 1-epimerase; 5.4.2.2 – phosphoglucomutase; 5.4.2.11 - 2,3-bisphosphoglycerate-dependent phosphoglycerate mutase; and 6.2.1.1 - acetyl-CoA synthetase.

A

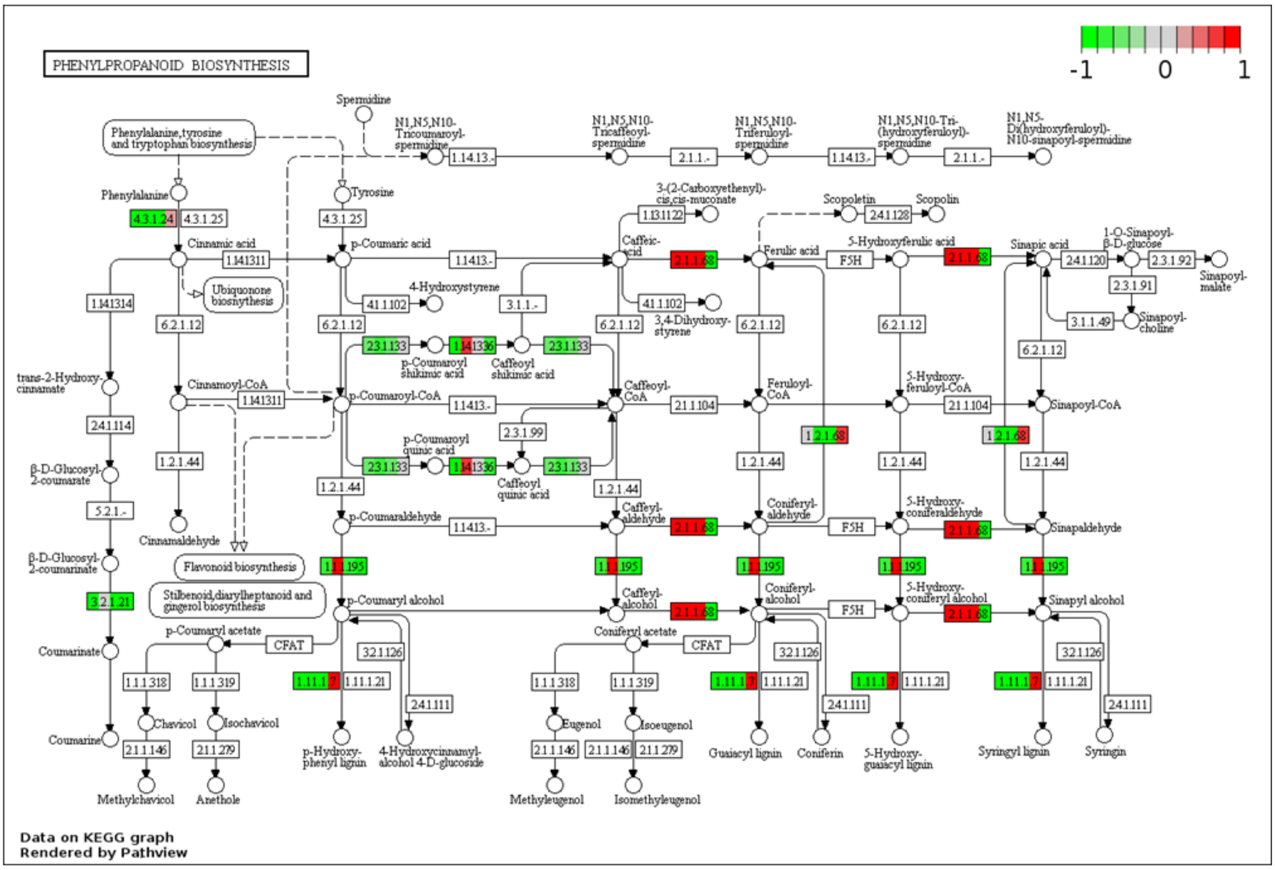
PHENYLPROPANOID BIOSYNTHESIS
-1
0
1
Phenylalanine,tyrosine and tryptophan biosynthesis
Phenylalanine
Tyrosine
Spermidine
Cinnamic acid
p-Coumaric acid
Caffeic acid
Ferulic acid
5-Hydroxyferulic acid
Sinapic acid
1-O-Sinapoyl-β-D-glucose
Sinapoyl-malate
Sinapoyl-choline
Scopoletin
Scopolin
Ubiquinone biosynthesis
Flavonoid biosynthesis
Stilbenoid,diarylheptanoid and gingerol biosynthesis
Cinnamoyl-CoA
p-Coumaroyl-CoA
Caffeoyl-CoA
Feruloyl-CoA
5-Hydroxy-feruloyl-CoA
Sinapoyl-CoA
Cinnamaldehyde
p-Coumaraldehyde
Caffeyl-aldehyde
Coniferyl-aldehyde
5-Hydroxy-coniferaldehyde
Sinapaldehyde
p-Coumaryl alcohol
Caffeyl-alcohol
Coniferyl-alcohol
5-Hydroxy-coniferyl alcohol
Sinapyl alcohol
p-Coumaryl acetate
Coniferyl acetate
Chavicol
Isochavicol
Methylchavicol
Anethole
Eugenol
Isoeugenol
Methyleugenol
Isomethyleugenol
p-Hydroxy-phenyl lignin
4-Hydroxycinnamyl-alcohol 4-D-glucoside
Guaiacyl lignin
Coniferin
5-Hydroxy-guaiacyl lignin
Syringyl lignin
Syringin
trans-2-Hydroxy-cinnamate
β-D-Glucosyl-2-coumarate
β-D-Glucosyl-2-coumarinate
Coumarinate
Coumarine
4-Hydroxystyrene
3,4-Dihydroxy-styrene
p-Coumaroyl shikimic acid
Caffeoyl shikimic acid
p-Coumaroyl quinic acid
Caffeoyl quinic acid
N1,N5,N10-Tricoumaroyl-spermidine
N1,N5,N10-Tricaffeoyl-spermidine
N1,N5,N10-Triferuloyl-spermidine
N1,N5,N10-Tri-(hydroxyferuloyl)-spermidine
N1,N5-Di(hydroxyferuloyl)-N10-sinapoyl-spermidine
3-(2-Carboxyethenyl)-cis,cis-muconate
4.3.1.24
4.3.1.25
1.14.13.11
1.14.13.-
1.14.13.14
6.2.1.12
2.4.1.114
5.2.1.-
3.2.1.21
1.2.1.44
2.3.1.133
1.14.13.36
4.1.1.102
3.1.1.-
2.3.1.99
1.13.11.22
2.1.1.68
2.1.1.104
2.4.1.128
2.4.1.120
2.3.1.92
2.3.1.91
3.1.1.49
F5H
1.1.1.195
CFAT
3.2.1.126
1.1.1.318
1.1.1.319
2.1.1.146
2.1.1.279
1.11.1.7
1.11.1.21
2.4.1.111
2.1.1.-
Data on KEGG graph
Rendered by Pathview

B

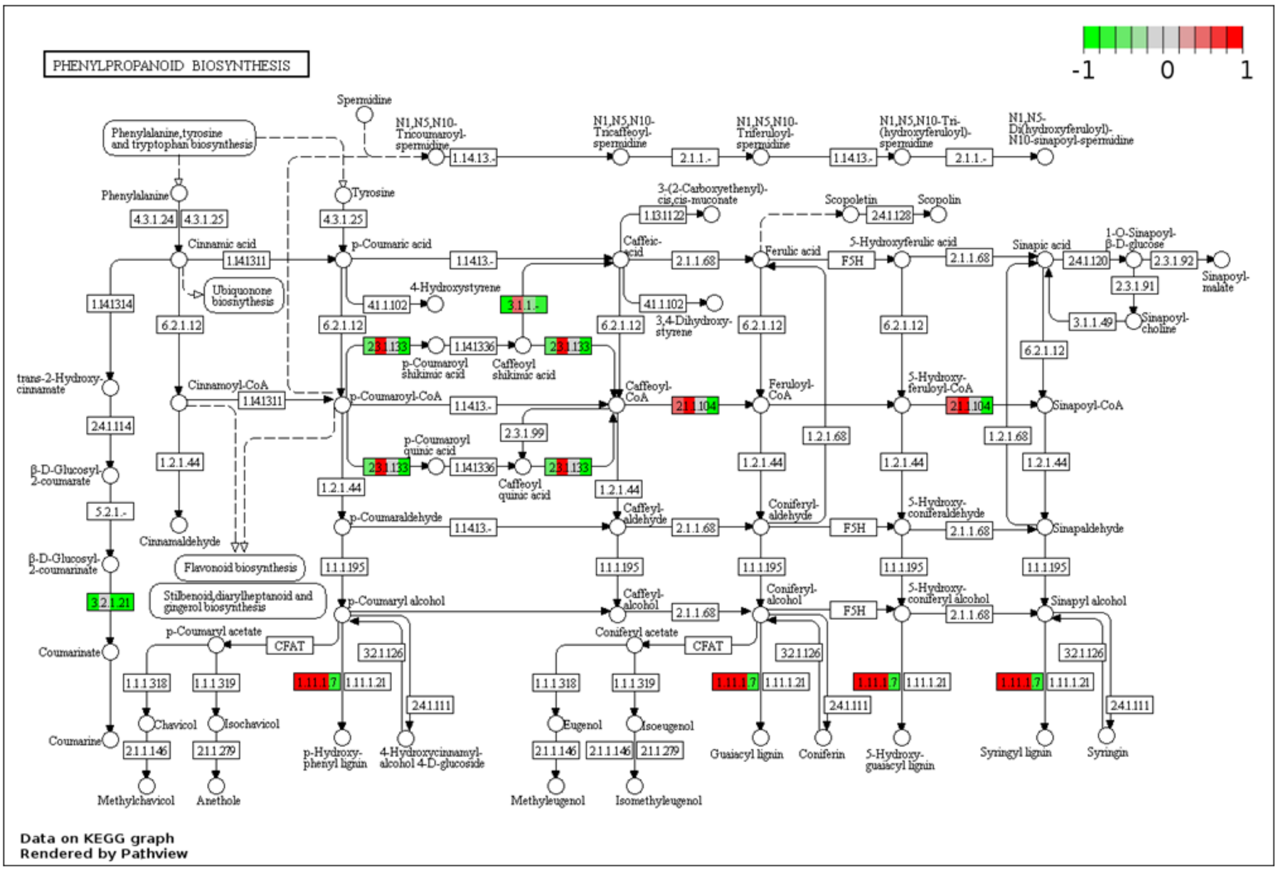

Figure S4. Differential transcript expression in the 'phenylpropanoid biosynthesis' KEGG pathway in the response to eCO_2 and Fe- limitation in root (a), and leaf tissues (b). Each pathway component contains four color bars indicating the mean expression of Fe+ELE/Fe+AMB (first), Fe-AMB/Fe+AMB (second), Fe-ELE/Fe+AMB (third), and Fe-ELE/Fe-AMB (fourth) treatments. Red color indicating increased expression, and green indicating decreased expression. 1.1.1.195 - cinnamyl-alcohol dehydrogenase; 1.2.1.68 - aldehyde dehydrogenase; 1.11.1.7 – peroxidase; 1.14.14.96 - cytochrome P450; 2.1.1.68 - caffeic acid 3-O-methyltransferase; 2.1.1.104 - caffeoyl-CoA O-methyltransferase; 2.3.1.133 - shikimate O-hydroxycinnamoyltransferase; 3.1.1.- - caffeoyl shikimate esterase; 3.2.1.21 - vicianin beta-glucosidase; and 4.3.1.24 - phenylalanine ammonia-lyase.
